# A Challenge From Longevity: Study on the Care Provider of the Chinese Oldest-Old at the End of Life

**DOI:** 10.1093/geroni/igaa057.1949

**Published:** 2020-12-16

**Authors:** Wenjuan Zhang

**Affiliations:** Renmin University of China, Beijing, China

## Abstract

The study aimed to investigate the changing pattern of care for the oldest-old at the end of life in China. Data were used from the Chinese Longitudinal Longevity survey from 1998 to2018. The results indicate significant changes in the care providers of older adults at the end of life from 1998 to 2018: grandchildren played an increasing important role in providing care to the oldest-old in China. The study highlights an important role of grandchildren who replace children as main caregivers. Longevity changes the traditional pattern of end-of-life care, and increase the diversity in the pattern of care for the Oldest-old, due to different life course of families compared with general aging families, and the limited role of formal social support system in providing end-of-life care for the oldest-old. We discuss implications in the context of increasing population of the oldest-old, and its challenges to formal social support system.

